# Sudden Death

**Published:** 1897-11

**Authors:** 


### SUDDEN DEATH

HELLER reported a case of sudden death in a healthy child. The child, which seemingly was perfectly well, only somewhat pale, had taken a drink just before its death with much enjoyment. The nurse had for a short time left the child, and found the latter dead on her return. The autopsy did not show the slightest cause to which death could be attributed. Dr. Cnopfsen, in the discussion which followed, mentioned the fact that in a child which suddenly died in his practice, an immense accumulation of fecal matter was found at the autopsy. It was his belief that death in these cases was due to autointoxication from the intestine, the products of putrefaction being absorbed from the intestine, and thus death induced.

---

No matter how great may be the ability of the practitioner as a diagnostician, the patient receives no advantage if the preparations administered are of indifferent quality; and if, as the result of substitution of imitation goods, the outcome of a given case is unsatisfactory, the physician will be blamed, not the druggist. See that your prescriptions are filled as written.

---

A writer in the *Presse Medicale* for June 19th, recommends the following mode of giving a vapor bath without removing the patient from bed: A woolen blanket is placed on the bed under the patient who keeps on his night robe. Under each foot and at each side of the body a stone bottle containing boiling water is placed, each bottle having previously been wrapped in a very wet towel and the whole covered with flannel. After the bottles are placed in position the woolen blanket is wrapped around the patient and another blanket and an eiderdown quilt are put over him.

At the end of fifteen minutes the patient is in a veritable vapor bath, which induces a profuse perspiration, and he is kept in this condition for a varying length of time according to the case. In order to favor sweating, one or two cupfuls of some hot infusion should be taken. After the patient has remained a sufficient length of time in the bath, the woolen blanket under him and the bottles are carefully withdrawn without exposing him, and he is then wiped dry under the other blanket and the quilt. At the end of twenty or thirty minutes the patient may have a change of linen.—*N. Y. Med. Journal.*

